# Ultrasonography can replace chest X-rays in the postoperative care of thoracic surgical patients

**DOI:** 10.1371/journal.pone.0276502

**Published:** 2022-10-20

**Authors:** Daniel J. Jakobson, Ornit Cohen, Evgenia Cherniavsky, Moris Batumsky, Lior Fuchs, Alon Yellin

**Affiliations:** 1 Intensive Care Department, Barzilai University Medical Center, Ashkelon, Israel; 2 Faculty of Health Sciences, Ben-Gurion University of the Negev, Beer-Sheba, Israel; 3 Faculty of Public Health Ben-Gurion University, Beer-Sheba, Israel; 4 Radiology Department, Barzilai University Medical Center, Ashkelon, Israel; 5 General Surgery Department, Barzilai University Medical Center, Ashkelon, Israel; 6 Intensive Care Department, Soroka University Medical Center, Beer Sheba, Israel; 7 Thoracic Surgery Department, Barzilai University Medical Center, Ashkelon, Israel; University of Texas Medical Branch at Galveston, UNITED STATES

## Abstract

**Objectives:**

Lung ultrasound accurately identifies pulmonary and pleural pathologies. Presently it has not been accepted as a routine examination in the postoperative follow-up of thoracic surgical patients. The present study aimed to compare thoracic ultrasonography with chest X-ray for detecting and clinical relevance of pneumothorax, pleural effusion, and lung consolidation and determine whether ultrasonography could replace chest X-ray as the standard examination after surgery.

**Methods:**

In this blinded, prospective, single-center study, lung ultrasound images were obtained within 2 hours of post-operative routine chest X-ray. A severity score was given to each examination in each technique. Lung ultrasound and chest X-ray results were compared by three methods: absolute comparison of normal to abnormal, the degree of pathology, and the clinical findings’ relevance.

**Results:**

Eighty patients were enrolled from 2013 to 2017, and 215 ultrasonography images were obtained. For pneumothorax, the precise overlap was found in 129/180 (72%) images. In 24% of examinations, X-ray missed ultrasonography findings. There was an agreement between studies in 80/212 (38%) images for pleural effusion. 60% of pleural effusions were missed by chest X-ray and detected by ultrasonography, and only 2.4% were missed by ultrasound, all very small. Clinically relevant fluid accumulation found a precise match in 80%, and 20% were found only by lung ultrasound. For lung consolidation, a 100% overlap was found with both methods.

**Conclusions:**

Our results suggest that lung ultrasound may replace chest X-ray as the standard examination in the postoperative care of patients undergoing thoracic surgical procedures.

## Introduction

Routine, daily or on-demand, chest X-rays (CXR) are part of the standard postoperative care of patients undergoing thoracic surgical procedures [1]. The main goal of the routine CXR is to evaluate the pleural space and help decide when chest tubes can be removed. CXRs are expensive, associated with potentially harmful radiation, utilize transportation resources, and are not available immediately.

Lung ultrasound (LU) has emerged as an excellent means in identifying various pleural and pulmonary pathologies [2]. It is very accurate in detecting pneumothorax (PNX) and pleural effusion (PE) and has been used as a tool for the removal of chest drains [3]. At the bedside, lung ultrasonography is highly sensitive, specific, and reproducible for diagnosing lung consolidations and PE and has higher diagnostic accuracy than auscultation and chest CXR [4].

Nevertheless, CXR is still the primary modality for post-operative lung assessment. We were able to find very few studies dedicated to the routine use of LU in the postoperative setting of thoracic surgical patients. A prospective study from 2012 had negative results [5]. That study was performed by inexperienced LU operators who probably ignored many US findings.

Based on the extensive experience we have gained in our intensive care unit, we hypothesized that a well-designed study would demonstrate that LU is non-inferior to CXR and can replace it in the management of patients undergoing thoracic surgery.

## Materials and methods

The institutional ethics committee approved the study (Approval No-0086-13-BRZ, 2nd October 2013). Consent of the patients was obtained before the surgical intervention.

The study was designed as a prospective blinded study performed in a 600 hundred-bed district hospital. For 42 months, patients scheduled to undergo an elective thoracic surgical procedure with a potential air leak at the Barzilai University Medical Center in Ashkelon, Israel, were enrolled in the study. Patients <18 years old or unable to consent were excluded. Written informed consent was obtained in the pre-operative clinic or during admission on the day preceding surgery.

Clinical decision-making was based solely on CXR, and the surgeon was blinded to LU results. Only patients, who had anatomical, or non-anatomical pulmonary resection with potential air-leak (formal decortication), were approved for the study. Most patients (72) had a single chest drain, and 8 (decortication cases) had two drains, all connected to a digital system and removed once the air-leak was smaller than 20 ml/min, the fluid discharge was less than 150 ml/24 hrs, and a CXR that did not demonstrate a significant pleural air space or effusion.

Our standard postoperative protocol required three routine CXRs, and an additional exam according to the symptoms. The first routine CXR was performed in the recovery room (supine position), the second in the ward before chest drain removal, and the last CXR in the radiology suite at least 4 hours following chest tube removal before discharge (standing).

A single surgeon performed all procedures. All LUs were performed and interpreted in real-time by one experienced ultrasound operator with over ten years of LU experience. One dedicated radiologist evaluated all CXRs. The ultrasonography operator and the radiologist were blinded to each other’s results.

**LU.** Only the operated hemithorax was evaluated. LU was performed within one hour from the first CXR in the recovery room. Subsequent LU examinations were performed every three hours from the CXR in the ward. All the examinations were performed using a Philips CX50 (Phillips medical systems, Andover, MA, USA) either with a linear array (L12-3) or a phased array (S5-1). Transducers were selected to adjust for patient body habitus and by performer preference. Focus and gain were adjusted to improve image quality using the automatic preset option (the iSCAN). The ultrasound field depth was adjusted to 3.5 times the pleura depth. The first LU examination after surgery was done in a recumbent position. Subsequent studies were done in a sitting position if tolerated or at supine 30° trunk elevation if the patient refused or was unable to sit. Analgesics before the examination were not prescribed.

Three pathologies were under scrutiny: pleural effusion (PE), pneumothorax (PNX), and consolidation.

### Ultrasound definitions of the finding

**PE** was defined by finding an anechoic area defined by regular borders and a variable inter-pleural distance showing a sinusoid pattern on M-mode [2]. In our post-operative patients, the fluid found was bloody, therefore more echogenic, and more challenging to identify [6] than regular non-hemorrhagic PE. **PNX** was annotated if lung sliding was absent, together with "A" pattern and stratospheric or bar-code sign observed at M-mode [7]. Identifying a lung point confirmed the diagnosis but was not mandatory for the diagnosis. **Consolidation** was noted when ultrasonographic examination showed an echogenic area with breath-dependent motion, air bronchogram, irregularly spaced lung comets (B lines), blurred or scattered margins [8,9].

To produce a standard comparison with the CXR, LU was done over six chest regions as described by Lichtenstein in his original study [10]: anterior (zone 1) defined by the anterior axillary line to the sternum, between the clavicle and the diaphragm; lateral (zone 2) between the anterior and posterior axillary lines between the axilla and the diaphragm; posterior (zone 3) flanked by the posterior axillary line to the spine, avoiding the scapula and down to the diaphragm. Each surveyed region was subdivided into superior and inferior areas, thus defining six areas for each examination. A severity score was given to each of the pathologies described. The absence of pathology in any region got a score of 0. The presence of any of the surveyed pathologies scored 1 for each zone, allowing a severity score of 0–6.

### Chest CXR definitions of the findings

Only the operated hemithorax was evaluated in the present study. The first CXR was done at supine 30° trunk elevation. Subsequent CXR was done on standing position.

**PE** was defined as small when the lateral costo-phrenic sinus was blurred or the para-costal line thickened; moderate when half of the hemithorax was obscured by fluid; severe when more than half of the hemithorax was covered by fluid and with signs of mass effect on mediastinal structures.

Estimation of **PNX** sizes was done according to BTS guidelines 2010 as: small or large [11].

#### Consolidation

was noted when alveolar opacity with indicting borders and air bronchogram were seen.

We analyzed the results in 3 different methods: a) The "absolute method" comparing normal to abnormal, defining LU score of no finding as 0, and the absence of any radiological finding as normal and any pathology as abnormal. b) The "precise method" where the degree of any pathology found by both techniques is compared. We defined three groups: normal, "minor" (LU score 1–2 and CXR’s small), and "major" (LU score 3–6, CXR’s large for PNX and moderate plus severe for PE). And c) The "clinically relevant method" where examinations are evaluated by their clinical relevance. For clinical purposes, the absence of PNX and PE, or only a mild degree of them, have the same effect, namely minimal impact and no need for intervention in a patient after thoracotomy with a chest tube. Thus normal examinations and those defined as "minor" (scores 0, 1, and 2) were grouped as clinically non-significant, and those defined as "major" (scores 3 to 6) were termed clinically significant. Each condition was analyzed by the three methods.

### Statistical methods

The primary outcome of this study was defined as a diagnosis of pleural effusion, lung consolidation, or pneumothorax. We aimed to address the diagnostic accuracy of CXR vs. LU for these conditions following thoracic surgery. Our a priori hypothesis was that LU would be equivalent to CXR in diagnosing the pathologies above. We based our sample size on a previous report by Lichtenstein et al., who described the comparative diagnostic performance of CXR and LU in ARDS [4]. In the mentioned report, LU and CXR had 93% and 47% sensitivity for diagnosing pleural effusion, respectively. Therefore, and accounting for a negligible difference margin of 10%, a two-sided alpha of 5%, and a power of 80%, we calculated a required sample size of at least 40 patients.

Baseline patient characteristics were obtained from a prospective database.

For statistical analysis, each examination was considered individually. Continuous parameters were expressed as mean ± standard deviation (SD), or as median and range, as appropriate. Categorical parameters were expressed as percentages. Spearman correlation and chi-square analysis were done in this study to assess the association between the two platforms, LU and CXR, by surgery day. For analysis purposes, each diagnosis in each modality was considered valid. SPSS (SPSS Inc., Chicago, Il, USA, version 24) was used to conduct all analyses. All statistical tests were 2-sided, and a p-value of less than 5% was considered to be statistically significant.

## Results

A total of 80 patients were enrolled in the study from November 2013 to April 2017, and 241 LU images were performed. Fifteen LU examinations were not included in the analysis because comparable CXRs were not performed. Eleven LU images were not included because the designated time window was breached. Thus, two hundred and fifteen examinations were available for evaluation. For scoring purposes, LU examinations in areas impeded by subcutaneous emphysema or large dressings were excluded from analysis, resulting in 180 examinations for the PNX category and 212 examinations for the PE category.

The study included 49 males and 31 females, 18–87 years old (mean 55.29, SD 18.05, median 58 years). The mean number of examinations per patient was 2.6 exams (median = 3, SD = 1, range 1–5). The mean number of days that the patient stayed after the operation was 6.4 days (SD = 6.1, median = 5, range 1–45). The mean time interval between the US and the comparative CXR acquisition was 46 ± 38 minutes.

LU was done within 60 minutes from the CXR in 95% of the cases and within 2 hours in 100%. The mean examination time required for the LU was 6.7±2.3 minutes (Range: 2–15, Median: 6 min). Only one agitated patient at the recovery room negated consent to the LU but agreed to participate in the subsequent days.

Indications for surgery were: Non-Small Cell Carcinoma (NSCLC)– 22 (27.5%), undiagnosed solitary lung mass– 17, pneumothorax– 17 (one traumatic), diffuse lung disease– 7, organizing empyema– 7, lung infections– 5, metastases– 2 and foreign body, giant bulla and malignant mesothelioma one each. The procedures consisted of simple or complex lobectomy (with chest wall resection or part of another lobe)– 33, segmentectomy– 2, decortication– 8, wedge resection– 36, mediastinal biopsy– 1. Sixty-two operations were performed by VATS and 18 via open thoracotomy. There were 17 complications: major– 11 (including eight patients with prolonged air-leak), minor– 5, and one death unrelated to the study intervention.

PNX: Analyzing the 180 images by the "absolute method," an agreement between LU and CXR was found in 129/180 (72%) (Table 1). Using the "precise method," a normal study was diagnosed by LU and CXR in 109 examinations (61%). Eighteen examinations (10%) were labeled "minor" by both. For the "major" category, two examinations (1%) correlated. In 43 cases (24%), LU identified PNX that CXR missed, and only in 8 patients (4%), LU missed PNX that were identified by CXR (Table 1). Analyzing the results by the "clinically oriented method" (Table 2), we found agreement in 169/180 examinations (94%), LU diagnosed in 11(6%) studies PNX that CXR missed, and missed none.

**Table 1:** PNX: The agreement between LU and CXRs by severity, the "precise method".

PNX						
			US			Total
			"0"	1–2	3–6	
x-ray	"0"	Count	109	32	6	147
		%	61%	18%	3%	82%
	1–2	Count	8	18	5	31
		%	4%	10%	3%	17%
	3–6	Count	0	0	2	2
		%	0.0%	0.0%	1%	1%
Total		Count	117	50	13	180
		%	65%	28%	7%	100.0%

PNX: Pneumothorax; US: Ultrasound; CXR: Chest X-ray; LU: Lung ultrasound.

**Table 2:** PNX: Agreement between LU and CXR by clinically oriented method (scores 0, 1–2 = non-significant; 3–6 = significant).

Pneumothorax				
		Ultrasound		
		Non-significant	Significant	Overall
Chest X-ray	Non-significant	167	11	178
	Significant	0	2	2
	Overall	167	13	180

PNX: Pneumothorax; US: Ultrasound; CXR: Chest X-ray; LU: Lung ultrasound.

PE: analyzing the 212 images by the "absolute method," an agreement was found in 80/212 (38%) of the images (Table 3). By the "precise method," LU identified PE that was missed by CXR in 127 cases (60%), and in 5 (2.4%), LU missed PE that was identified by CXR (Table 3). Shifting to the "clinically oriented method" (Table 4), we found agreement in 170 examinations (80%); in 43 (20%), only LU identified PE, and LU missed PE in none.

**Table 3:** PE: The agreement between LU and CXRs by severity, the "precise method".

PE						
			US			Total
			"0"	1–2	3–6	
x-ray	"0"	Count	59	85	21	165
		%	29%	40%	10%	77.8%
	1–2	Count	5	20	21	46
		%	2%	9%	10%	21.7%
	3–6	Count	0	0	1	1
		%	0.0%	0.0%	0.5%	0.5%
Total		Count	64	105	43	212
		%	31%	49%	20.5%	100.0%

PE: Pleural effusion; US: Ultrasound; CXR: Chest X-ray; LU: Lung ultrasound.

**Table 4:** PE: Agreement between LU and CXR by clinically oriented method (scores 0, 1–2 = non-significant; 3–6 = significant).

Pleural Effusion				
		Ultrasound		
		Non-significant	Significant	Overall
Chest X-ray	Non-significant	169	42	211
	Significant	0	1	1
	Overall	169	43	212

PE: Pleural effusion; US: Ultrasound; CXR: Chest X-ray; LU: Lung ultrasound.

Consolidation: In both the "absolute method" and the "precise method,” agreement was found for all the 38 cases (100%) over the 215 compared images (Table 5). The “clinically oriented method" found a complete correlation," where all the 30 cases were minor.

**Table 5:** Consolidation: The agreement between LU and CXRs.

		CXRs		
		Normal	Pathology	Total
LU	Normal	177 (82%)	0 (0%)	177 (82%)
	Pathology	0 (0%)	38 (18%)	38 (18%)
	Total	177 (82%)	38 (18%)	215 (100%)

## Discussion

Our study confirms that LU is a very sensitive tool in detecting pleural and pulmonary changes after thoracic surgery. The two main pathologies sought in the postoperative period are pleural effusion (PE) and pneumothorax (PNX). Previous studies have shown that LU has a higher sensitivity than CXR in detecting both conditions [7,12,13]. Nevertheless, the standard study post thoracic surgery is CXR [1], and therefore LU findings were compared to it and not vice versa. Missed findings by CXR that were diagnosed by LU, or CXR findings that LU did not confirm, may be considered by surgeons as false positive or false negative results, respectively. A similar study on patients undergoing cardiac surgery was reported recently [14]. It showed that US was superior to CXR in detecting overall and clinically relevant postoperative pulmonary complications.

Determining absolute changes (0 Vs. any degree of PE or PNX) resulted in a significant mismatch. Using such a crude method has no clinical significance as the "positive" group may include cases with minimal findings along with those with significant findings. We, therefore, analyzed by the precise method, but this again seemed to be clinically useless because it creates a 3X3 table with huge but clinically insignificant findings by LU that CXR missed. Only the clinically relevant method had a very high agreement rate and seemed to serve as a decision-making tool.

We have no concern over cases that LU only diagnosed because for these cases, most probably, a CXR would be ordered for confirmation. The 8 (4%) LU missed PNXs are more bothersome. Still, studying each of these cases carefully noted that they all occurred in the recovery room examinations. None had clinical complications and required a change of management. On sequential tests, no pneumothorax was missed. Possible explanations for missing them include: PNX restricted to areas challenging to study by LU, subcutaneous emphysema that prevented an optimal LU examination, and interpreter’s error. LU detected 63 PNXs compared to 33 observed by CXR. This demonstrates the actual superiority of LU over CXR in PNX diagnosis [11,12].

The sensitivity of LU in the detection of PE is well known [15,16]. In our study, three-fold more PEs were seen on LU than CXR, while LU missed no case. If CXR is the accepted gold standard in thoracic surgery post-op monitoring, it appears as if LU has a substantial false-positive rate. Nevertheless, according to previous LU studies, the results probably reflect the limitations of CXR to detect small PEs, especially in the supine postoperative patient. The first CXR performed in a supine position has a high threshold for PE and is inaccurate in detecting small PNXs [7].

On the other hand, LU can detect PE as small as 20 ml [17]. Considering the very high sensitivity of LU in detecting PNX and PE, it seems that new definitions for "normal" and "abnormal" are required in the setting of the postoperative patient. For this purpose, we used the clinical relevance method, which seems more suitable for this setting and would significantly decrease the need for CXRs. One must remember: a small finding can become larger, clinically insignificant finding can become significant. Clinicians should defer knowing if there is a small PE or a small PNX rather than not knowing.

Although not a primary goal of the current study, extraordinary results were observed for consolidation. Both CXR and LU detected the same 38 minor findings with a 100% match. As these patients had no additional signs of infection, the consolidations mentioned above probably represent areas of parenchymal contusion secondary to surgical manipulations.

The mean examination time required for the LU was 6.7±2.3 minutes (Range: 2–15, Median: 6 min). A mean time of 2.3±2.9 minutes was reported previously for the ultrasonographic diagnosis of a pneumothorax [18]. The longer time in our study originated from the fact that we searched for three possible pleural and parenchymal conditions and examined the hemithorax at six separate areas. The comparative time to get the CXR examination done and interpreted at one study for pneumothorax diagnosis was 19.9±10.3 minutes [18].

Our study has some limitations. Although we did not measure pain in the current study, one would not expect more discomfort from slightly displacing the arm to reach all points of the LU compared to that generated by sliding the CXR grid below the patient’s body. To our experience, the first CXR after thoracic surgery is very painful for patients. Remarkably, the only patient who refused LU in the post-operative care unit had a CXR as part of a mandatory post-operative routine and was done without asking for consent. As mentioned in the method section, we did not administer extra narcotics or analgesics before the LU examinations. The pain scores used routinely in our practice (though not analyzed in detail) were grossly unchanged.

LU requires an operator competent in achieving the correct methodology needed for a comprehensive study. Ideally, the operator is also the interpreter. The experience of the operator may have played a role in previous studies. The operator in our study was highly experienced. Lack of experience may have contributed to the negative results in the study by Goudie et al. [5]. We believe that in the near future, every thoracic surgeon will master this tool and be able to interpret the results as he does with CXR and CT.

Although LU is superior to CXR in detecting several thoracic pathologies, it is hindered in the presence of significant subcutaneous emphysema. Small amounts of air in the chest wall can be dealt with by pressing the transducer over the examined area and waiting for air bubbles to be displaced [19]. Still, the obtained image can be inappropriate for clinical decision-making, and large subcutaneous emphysema would make the study useless and necessitate a CXR. In our study, only one examination was performed in the ICU (was unable to demonstrate PNX in the presence of significant subcutaneous emphysema.

Another limitation to our study is the low rate of complications observed that interfere with the clinical interpretation of the results. In addition, the fact that the study was terminated prematurely can potentially affect the results, although statistically, they were strong enough to reach significant conclusions. We may be criticized for performing LU only on the operated hemithorax. Still, one must appreciate that the primary goal of routine CXRs is to determine whether the pleura is adequately drained and when the chest tubes could be removed. LU, as studied presently, is relevant only in comparison to the routine CXRs performed after thoracic surgical procedures. It does not obviate the need for other studies like CT or CTA when the clinical course calls for them. Considering the short time needed for a complete lung ultrasound, we recommend establishing two lungs scan rather than the one lung scan we have done for a complete lung imaging. This information will be more comparable to the standard post-operative CXR.

Lastly, although our research was conducted on adults and cannot be generalized to all patients, recent reports from a clinical research program where serial LUs were routinely performed after pediatric cardiac surgery found several advantages of LU over radiography in assessing postoperative pulmonary complications. They discovered that LU was feasible in all patients, significantly reduced costs and radiation exposure [20], and was more accurate than CXR in diagnosing pleural effusion atelectasis and lung congestion [20]. Also, LU score in cardiac surgery pediatric patients added to the prognosis of different ICU clinical outcomes [21].

## Conclusions

Our study suggests that lung ultrasound may effectively replace CXR in most patients undergoing thoracic surgery routine follow-up. Larger studies are needed to confirm our findings. We believe that lung ultrasound will become the method of choice for the postoperative evaluation of thoracic surgical patients.
